# Novel Compound Heterozygous *PRKN* Variants in a Han-Chinese Family with Early-Onset Parkinson's Disease

**DOI:** 10.1155/2019/9024894

**Published:** 2019-12-23

**Authors:** Kuan Fan, Pengzhi Hu, Chengyuan Song, Xiong Deng, Jie Wen, Yiming Liu, Hao Deng

**Affiliations:** ^1^Center for Experimental Medicine, The Third Xiangya Hospital, Central South University, Changsha, China; ^2^Department of Neurology, Guizhou Provincial People's Hospital, Guiyang, China; ^3^Department of Radiology, The Third Xiangya Hospital, Central South University, Changsha, China; ^4^Department of Neurology, The Qilu Hospital, Shandong University, Jinan, China; ^5^Department of Neurology, The Third Xiangya Hospital, Central South University, Changsha, China

## Abstract

Genetic factors are thought to play an important role in the pathogenesis of Parkinson's disease (PD), particularly early-onset PD. The *PRKN* gene is the primary disease-causing gene for early-onset PD. The details of its functions remain unclear. This study identified novel compound heterozygous variants (p.T240K and p.L272R) of the *PRKN* gene in a Han-Chinese family with early-onset PD. This finding is helpful in the genetic diagnosis of PD and also the functional research of the *PRKN* gene.

## 1. Introduction

Parkinson's disease (PD) is the second most frequent neurodegenerative disease after Alzheimer's disease [1]. Parkinsonism, the core clinical feature of PD, is defined as slowly progressive bradykinesia combined with rest tremor or rigidity [2]. The etiology of PD remains enigmatic, while environmental and genetic factors are thought to be involved in [3]. Currently, 23 disease-causing loci and 19 genes have been identified for PD and recorded in the Online Mendelian Inheritance in Man (OMIM) [4]. Although PD mainly affects those over 50, early-onset PD (EOPD) patients, whose motor disorder symptoms appear before age 40, account for 3–5% of all PD patients worldwide [5]. EOPD's primary genetic type is autosomal recessive juvenile parkinsonism (AR-JP, OMIM 600116) caused by homozygous or compound heterozygous mutations in the parkin RBR E3 ubiquitin protein ligase gene (*PRKN*) [6]. It has parkinsonism symptoms, but may be a different disease entity from late-onset sporadic PD. This is suggested by more dystonia at onset, better levodopa responsiveness, slower disease progression, sleep benefit, and lower frequencies of nonmotor symptoms and Lewy bodies [7–9].

**Figure 1:**
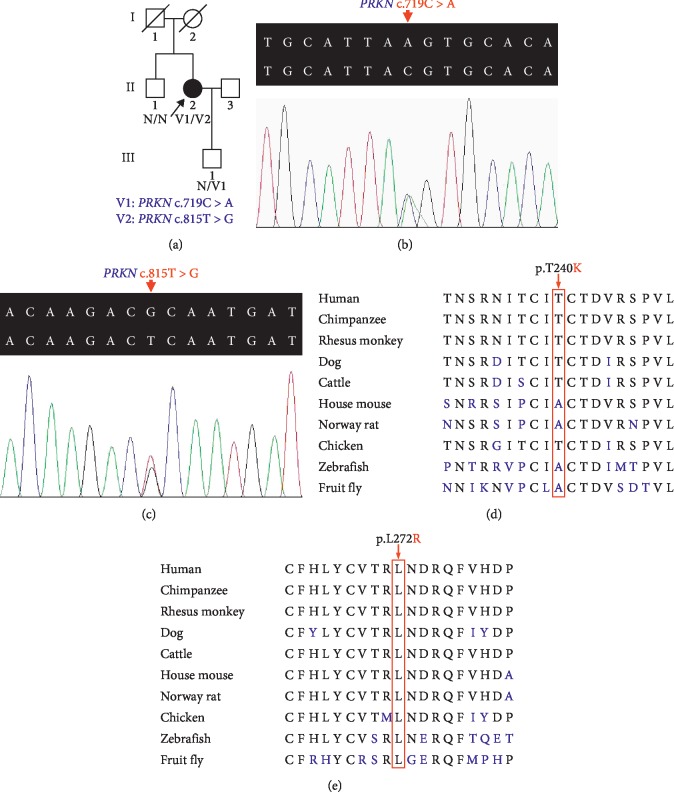
(a) Pedigree of the family with EOPD. The arrow indicates the proband. (b) DNA sequencing of the c.719C > A variant in the *PRKN* gene. (c) DNA sequencing of the c.815T > G variant in the *PRKN* gene. (d) Conservation analysis of the parkin p.T240 amino acid residue. (e) Conservation analysis of the parkin p.L272 amino acid residue.

This gene encodes the parkin protein which could ubiquitinate numerous mitochondrial outer membrane proteins resulting in autophagy of damaged mitochondria, and *PRKN* mutations caused mitochondrial quality control deficiencies and neuron death [10]. The parkin protein has been intensely studied due to its complex activation mechanisms and suppressive roles in various tumors [11, 12]. This study reports on new compound heterozygous variants in the *PRKN* gene in a family with EOPD (Figure 1). This finding expands the *PRKN*-associated PD genetic spectrum and may provide new insights into parkin protein structures and functions.

## 2. Materials and Methods

A Han-Chinese family with EOPD was recruited from China's Shandong Province (Figure 1(a)). The proband, her brother, and son were examined and diagnosed by two experienced neurologists. Two hundred unrelated healthy Han-Chinese older than 40 were enrolled as controls. This study was approved by the Institutional Review Board of the Third Xiangya Hospital of Central South University, Changsha, Hunan, China. After written informed consents were obtained, peripheral venous blood samples were taken from all participants. gDNA isolated from the proband (II : 2) was target captured by a PD-associated gene panel. Subsequently, paired-end sequencing using Illumina HiSeq X-ten platform (Illumina Inc., USA) was performed. All potential variants were filtered according to the Single Nucleotide Polymorphism database, the 1000 Genomes Project, and the Exome Aggregation Consortium database. Coding indels, potential splice-site changes, and nonsynonymous single-nucleotide variants in exons with minor allele frequency <10^−3^ were considered as pathogenic candidates. Sanger sequencing verified candidate variants using an ABI3500 sequencer (Applied Biosystems Inc., USA) [13]. Sorting Intolerant from Tolerant (SIFT) and Combined Annotation Dependent Depletion (CADD) predicted the potential pathogenic effects of variants. The Basic Local Alignment Search Tool analyzed amino acid sequence conservations. The methods of targeted sequencing and Sanger sequencing are detailed in the Supplementary Materials.

## 3. Results

### 3.1. Clinical Features

The age at onset of the proband (II : 2) was 32 years. The initial symptoms were slowness and rest tremor in her right arm, and tightness appeared one year later. These motor symptoms slowly progressed and spread to the right leg and contralateral limbs over a period of six years. Levodopa therapy significantly improved motor symptoms as of her first examination at age 35 years, but detailed evaluation was not performed. At her latest evaluation, at age 39 years, clinically established PD was diagnosed on the basis of prominent bradykinesia symptom and other PD-related symptoms, including rest tremor, rigidity, face masking, numbness, difficulty falling asleep, olfactory impairment, constipation, mild cognitive impairment, depression, and anxiety. Her MDS Unified Parkinson's Disease Rating Scale motor score was 9 (on)/14 (off), and Mini-Mental State Examination score was 22. Skull CT and MRI results, blood and urine copper levels, and ceruloplasmin were all normal. No movement disorders or other nervous system disorders were found in her son (III : 1) or older brother (II : 1).

### 3.2. Molecular Findings

After target capture sequencing and filtering, only the p.T240K (c.719C > A, rs137853054) and p.L272R (c.815T > G) variants of *PRKN* gene were considered as pathogenic candidates in the known monogenic PD-causing genes. Subsequent Sanger sequencing confirmed both in the proband (Figures 1(b) and 1(c)). The p.T240K variant was found in her son (III : 1). The two variants were absent from her older brother (II : 1) and from the 200 normal controls. The p.T240K variant has a very low recorded heterozygous state frequency in the Genome Aggregation Database (gnomAD, 7.954 × 10^−6^). SIFT and CADD predicted the c.719C > A (p.T240K) variant as damaging (Table 1). The p.L272R variant has not been reported in the gnomAD and also has a damaging prediction in SIFT and CADD analysis (Table 2). Multisequence alignment shows that the leucine at position 272 is phylogenetically conserved from fruit flies to humans (Figure 1(e)). These data indicate that the compound heterozygous variants, p.T240K and p.L272R, are probably disease-causing for EOPD in this family.

## 4. Discussion

**Figure 2:**
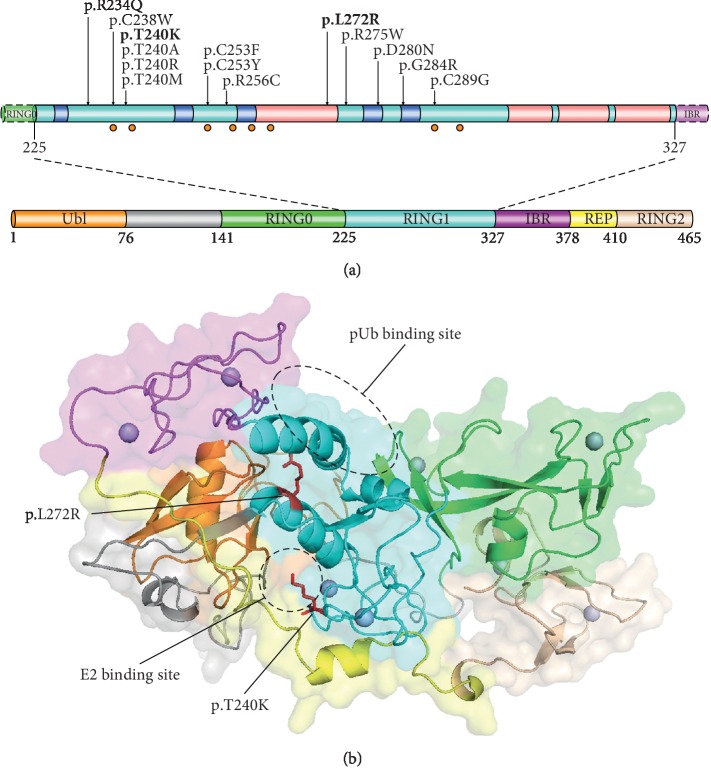
(a) Domain structure and missense variants associated with autosomal recessive parkinsonism detected in the RING1 domain of parkin protein [10, 14, 15, 21, 23–28]. Two variants detected in this study are highlighted in bold. *β*-Strand and *α*-helix are in dark blue and pink, respectively. Zinc-binding cysteines are signed with orange dots. (b) Cartoon representation of the p.T240K and p.L272R variants in parkin protein by PyMOL. Parkin protein crystal structure (PDB code 5C1Z) was used as a template [29]. Variants are shown as red stick models, and Zn^2+^ are shown as spheres. pUb, Ser65-phosphorylated ubiquitin.

The parkin protein is a 465-amino acid E3 ubiquitin ligase of the RING-between-RING (RBR) family, which could catalyse the transfer of ubiquitin from the E2 conjugating enzyme to substrate proteins [10]. Numerous recent studies attempted to determine the relationship between the parkin protein structure and its functions through *in vitro* and animal experiments. The most direct and compelling evidence for protein structure change effects remains the mutations, particularly missense mutations detected in patients. Approximately 25% of all *PRKN* gene mutations have been found in the RING1 domain, which is considered to have a binding site for the E2 conjugating enzyme and be important to parkin activation due to its interactions with Ser65-phosphorylated ubiquitin and the UbL domain (Figure 2) [10, 14, 15, 21–29]. The detailed molecular structure of parkin remains unclear, which results in several different activation and catalysis models [30].

In this study, clinically established EOPD and extensive nervous system impairment were suggested by the presence of three cardinal motor symptoms and multiple nonmotor symptoms in the proband. Two missense variants (p.T240K and p.L272R) with potential pathogenicity are located in the RING1 domain and had not been previously reported in PD patients. Although the sequence conservation of threonine at position 240 is lower than that of leucine at position 272 (Figures 1(d) and 1(e)), there have been at least 16 reported PD patients with *PRKN* p.T240M, p.T240R, or p.T240A variants in homozygous or compound heterozygous states (Table 1) [6, 14–21]. This amino acid is in the first Zn-binding loop of the RING1 domain which has been regarded as the E2 binding site of parkin [31]. The p.T240R mutation has been found to change the E2 binding interface and destroy the autoubiquitination activation of parkin protein and lead to EOPD [31, 32]. The first *α*-helix (260–273) of the RING1 domain was reported as the binding site for the UbL domain, which adjoins the RING1 domain through hydrophobic interaction to block E2 access [10, 33]. Three other missense variants (p.L272I, p.L272V, and p.L272F) and one synonymous variant in the 272nd codon have been recorded. However, no pathogenic evidence has been reported for these four variants, which may be due to low allele frequencies in generating populations and preservation of hydrophobicity (Table 2). The p.L272R, resulting in materially hydrophilic alternation, is more likely to disrupt protein folding and affect its function, especially in a compact protein stabilized by numerous hydrophobic interactions such as parkin (Table 2) [34, 35]. Investigating the structural changes resulting from the p.L272R variant may contribute to understanding parkin domain interactions and their potential function. More evidence including segregation information and functional research is needed to classify the two variants as pathogenic or likely pathogenic variants according to the American College of Medical Genetics and Genomics guidelines for variants interpretation [36].

In conclusion, the novel compound heterozygous variants of *PRKN* gene, p.T240K and p.L272R, were identified as the probable genetic cause for EOPD in a family with clinically established EOPD. These two missense variants both lead to amino acid changes in the RING1 domain. This finding has potential value for functional research of the *PRKN* gene and genetic diagnosis of PD. Further studies are warranted to clarify their pathogenicity and may offer deeper understanding of the detailed functional effects.

## Figures and Tables

**Table 1:** Reported variants in the 240th codon of the *PRKN* gene.

Nucleotide change	Amino acid change	Identifier	MAF (gnomAD)	SIFT		CADD score^a^	Reported patients^b^
				Score	Prediction		
**c.719C** **>** **A**	**p.T240K**	**rs137853054**	**7.954** **×** **10**^**−6**^	**0.01**	**Damaging**	**23.6**	**1**
c.719C > T	p.T240M	rs137853054	3.465 × 10^−4^	0.00	Damaging	23.8	13 [6, 14–20]
c.719C > G	p.T240R	—	—	0.00	Damaging	23.4	1 [21]
c.718A > G	p.T240A	—	—	1.00	Tolerated	14.97	2 [15]
c.718A > T	p.T240S	rs1194371893	7.954 × 10^−6^	0.03	Damaging	21.8	—
c.720G > A	p.T240=	rs769882260	3.536 × 10^−5^	—	—	5.479	—
c.720G > C	p.T240=	rs769882260	3.977 × 10^−6^	—	—	4.599	—

MAF, minor allele frequency; gnomAD, Genome Aggregation Database; SIFT, Sorting Intolerant from Tolerant; CADD, Combined Annotation Dependent Depletion. ^a^PHRED-scaled CADD score. ^b^Reported patients with *PRKN* variants in homozygous or compound heterozygous states.

**Table 2:** Reported variants in the 272nd codon of the *PRKN* gene.

Nucleotide change	Amino acid change	Identifier	MAF (gnomAD)	Hydropathy index	SIFT		CADD score^a^	Reported patients^b^
					Score	Prediction		
**c.815T** **>** **G**	**p.L272R**	—	—	**−4.5**	**0.00**	**Damaging**	**29.2**	**1**
c.814C > T	p.L272F	rs141366047	3.980 × 10^−6^	2.8	0.00	Damaging	25.6	—
c.814C > G	p.L272V	rs141366047	1.194 × 10^−5^	4.2	0.05	Damaging	24.4	—
c.814C > A	p.L272I	rs141366047	9.553 × 10^−5^	4.5	0.01	Damaging	25.1	—
c.816C > T	p.L272 =	rs143902760	1.322 × 10^−3^	3.8	—	—	9.553	—

MAF, minor allele frequency; gnomAD, Genome Aggregation Database; SIFT, Sorting Intolerant from Tolerant; CADD, Combined Annotation Dependent Depletion. ^a^PHRED-scaled CADD score. ^b^Reported patients with *PRKN* variants in homozygous or compound heterozygous states.

## Data Availability

The data used to support the findings of this study are available from the corresponding author upon request.

## Supplementary Materials

Supplementary Materials The detailed methods of targeted sequencing and Sanger sequencing used in this study are included.
