# Supplementary material for: Novel Compound Heterozygous PRKN Variants in a Han-Chinese Family with Early-Onset Parkinson's Disease
Source: Parkinsons Dis. 2019 Dec 23;2019:9024894. doi: 10.1155/2019/9024894 (PMC6942881; doi:10.1155/2019/9024894)
Supplement: Supplementary Materials — The detailed methods of targeted sequencing and Sanger sequencing used in this study are included. [file 9024894.f1.doc]

**Supplementary Materials.**

Targeted sequencing

The gDNA from the proband (II:2) was sonically fragmented and target captured by a panel containing all coding exons of 127 PD- or other dystonia diseases-associated genes (RunningGene Inc., Beijing, China). Subsequently paired-end sequencing using Illumina HiSeq X-ten platform (Illumina Inc., San Diego, CA, USA) was performed. A mean coverage depth of more than 100× and more than 95% of the target regions having at least 20× coverage were required to guarantee sequencing accuracy. Cutadapt (https://pypi.python.org/pypi/cutadapt) and FastQC (www.bioinformatics.babraham.ac.uk/projects/fastqc/) were performed for quality control. Clean reads were mapped to the human reference genome sequence from the UCSC database (version hg19, http://genome.ucsc.edu/) using Burrows-Wheeler Alignment (version 0.7.10). Variants were detected by GATK (version 3.1) and annotated using ANNOVAR software (version 2015Dec14).

Sanger sequencing

Sanger sequencing verified candidate variants using an ABI3500 sequencer (Applied Biosystems Inc., Foster City, CA, USA). Primer sequences were as follows: p.T240K: 5’-CCAAAGAGATTGTTTACTGTGGAA-3’ and 5’-GCTCGTGTGGCAGAACAATA-3’; p.L272R: 5’-GTGCTGCCTTTCCACACTG-3’ and 5’-CCTTCATTCCCCAGAACTTTT-3’.
